# Supplementary material for: Five Amino Acid Substitutions in the S1 Unit of Infectious Bronchitis Virus Are Critical Determinants Enhancing Its Adaptation to Vero Cells
Source: Vet Sci. 2025 Apr 22;12(5):394. doi: 10.3390/vetsci12050394 (PMC12115792; doi:10.3390/vetsci12050394)
Supplement: Supplementary file 1 [file vetsci-12-00394-s001.zip › vetsci-3562622-supplementary.pdf]

```
EP3(AAY24423.1)     MLVTPLLLVTLLCALCSAVLYDSSSYVYYYQSAFRPPNGWHLHGGAYAVVNISSESNNAG     60
P65(AAY24433.1)     MLVTPLLLVTLLCALCSAVLYDSSSYVYYYQSAFRPPSGWHLQGGAYAVVNISSEFNNAG     60
                    *************************************.****:************ ****
EP3(AAY24423.1)     SSPGCIVGTIHGGRVVNASSIAMTAPSSGMAWSSSQFCTAHCNFSDTTVFVTHCYKYDGC     120
P65(AAY24433.1)     SSSGCTVGIIHGGRVVNASSIAMTAPSSGMAWSSSQFCTAHCNFSDTTVFVTHCYKHGGC     120
                    ** ** ** ***********************************************:.**
EP3(AAY24423.1)     PITGMLQKNFIRVSAMKNGQLFYNLTVSVAKYPTFKSFQCVNNLTSVYLNGDLVYTSNET     180
P65(AAY24433.1)     PITGMLQQNFIRVSAMKNGQLFYNLTVSVAKYPTFRSFQCVNNLTSVYLNGDLVYTSNET     180
                    *******:***************************:************************
EP3(AAY24423.1)     TDVTSAGVYFKAGGPITYKVMREVKALAYFVNGTAQDVILCDGSPRGLLACQYNTGNFSD     240
P65(AAY24433.1)     IDVTSAGVYFKAGGPITYKVMREVKALAYFVNGTAQDVILCDGSPRGLLACQYNTGNFSD     240
                     ***********************************************************
EP3(AAY24423.1)     GFYPFINSSLVKQKFIVYRENSVNTTFTLHNFTFHNETGANPNPSGVQNIQTYQTQTAQS     300
P65(AAY24433.1)     GFYPFTNSSLVKQKFIVYRENSVNTTCTLHNFIFHNETGANPNPSGVQNIQTYQTKTAQS     300
                    ***** ******************** ***** **********************:****
EP3(AAY24423.1)     GYYNFNFSFLSSFVYKESNFMYGSYHPSCNFRLETINNGLWFNSLSVSIAYGPLQGGCKQ     360
P65(AAY24433.1)     GYYNFNFSFLSSFVYKESNFMYGSYHPSCKFRLETINNGLWFNSLSVSIAYGPLQGGCKQ     360
                    *****************************:******************************
EP3(AAY24423.1)     SVFSGRATCCYAYSYGGPSLCKGVYSGELDLNFECGLLVYVTKSGGSRIQTATEPPVITQ     420
P65(AAY24433.1)     SVFKGRATCCYAYSYGGPSLCKGVYSGELDHNFECGLLVYVTKSDGSRIQTATEPPVITQ     420
                    *** ************************** *************.***************
EP3(AAY24423.1)     HNYNNITLNTCVDYNIYGRTGQGFITNVTDSAVSYNYLADAGLAILDTSGSIDIFVVQGE     480
P65(AAY24433.1)     HNYNNITLNTCVDYNIYGRTGQGFITNVTDSAVSYNYLADAGLAILDTSGSIDIFVVQGE     480
                    ************************************************************
EP3(AAY24423.1)     YGLNYYKVNPCEDVNQQFVVSGGKLVGILTSRNETGSQLLENQFYIKITNGTRRFRRSIT     540
P65(AAY24433.1)     YGLNYYKVNPCEDVNQQFVVSGGKLVGILTSRNETGSQLLENQFYIKITNGTRRFRRSIT     540
                    ************************************************************
```

Figure S1. Amino acid alignment of the S1 region for both IBV-P65 and IBV-EP3. The amino acid changes are highlighted in boldface. “*”, fully conserved residue; “:”, conserved mutation; “.”, semi-conserved mutation.
