# Supplementary material for: Five Amino Acid Substitutions in the S1 Unit of Infectious Bronchitis Virus Are Critical Determinants Enhancing Its Adaptation to Vero Cells
Source: Vet Sci. 2025 Apr 22;12(5):394. doi: 10.3390/vetsci12050394 (PMC12115792; doi:10.3390/vetsci12050394)
Supplement: Supplementary file 1 [file vetsci-12-00394-s001.zip › vetsci-3562622-WB figure.pdf]

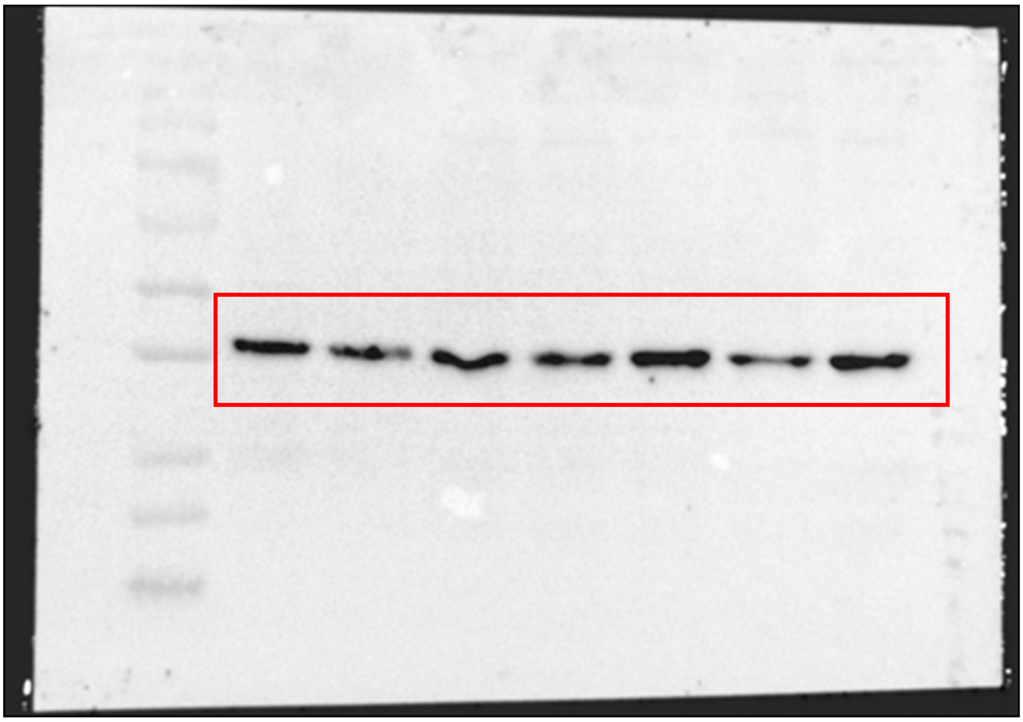

Uncropped Western blotting images. 16h Actin protein (Marked with red square).

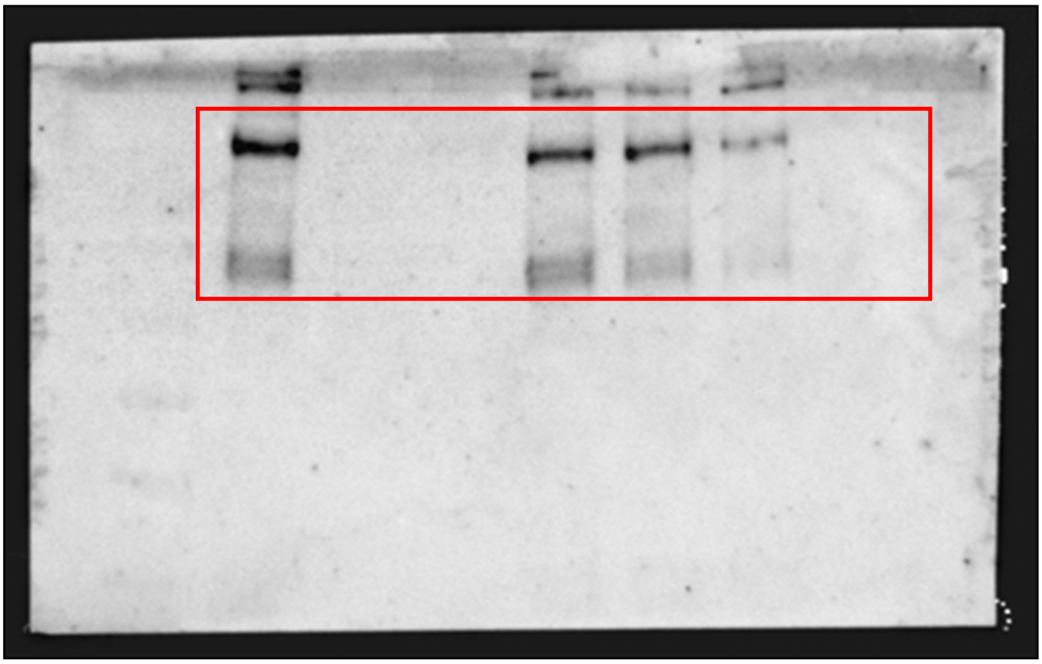

Uncropped Western blotting images. 16h S protein (Marked with red square).

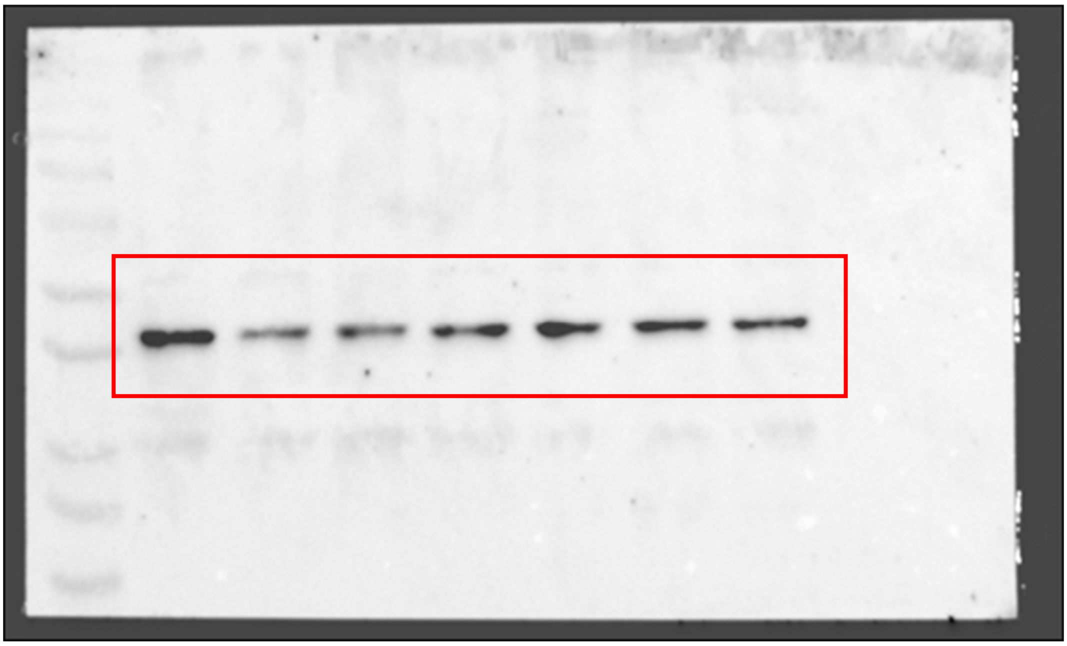

Uncropped Western blotting images. 24h Actin protein (Marked with red square).

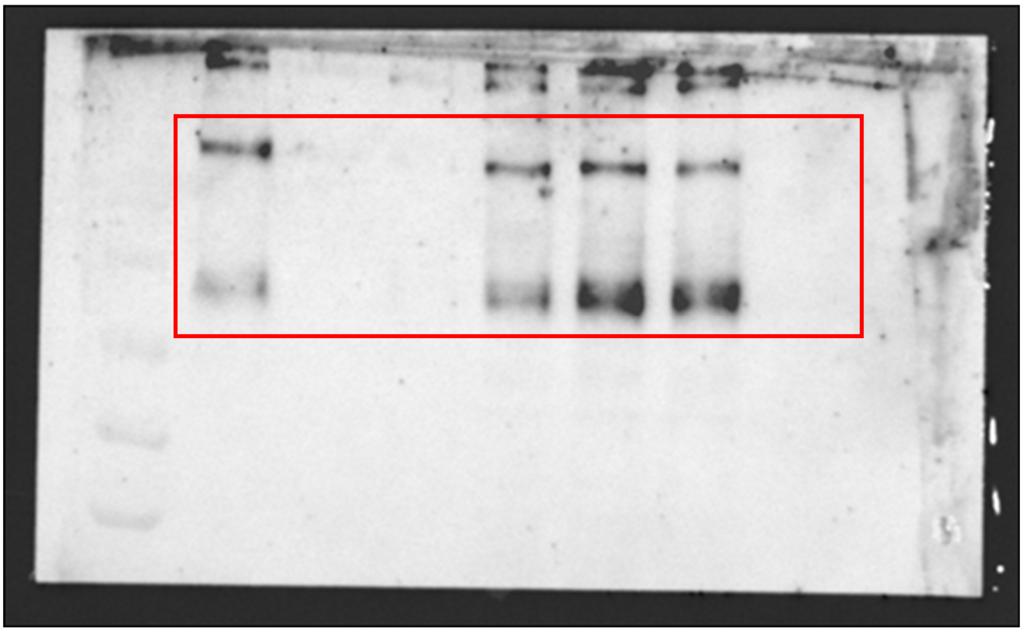

Uncropped Western blotting images. 24h S protein (Marked with red square).

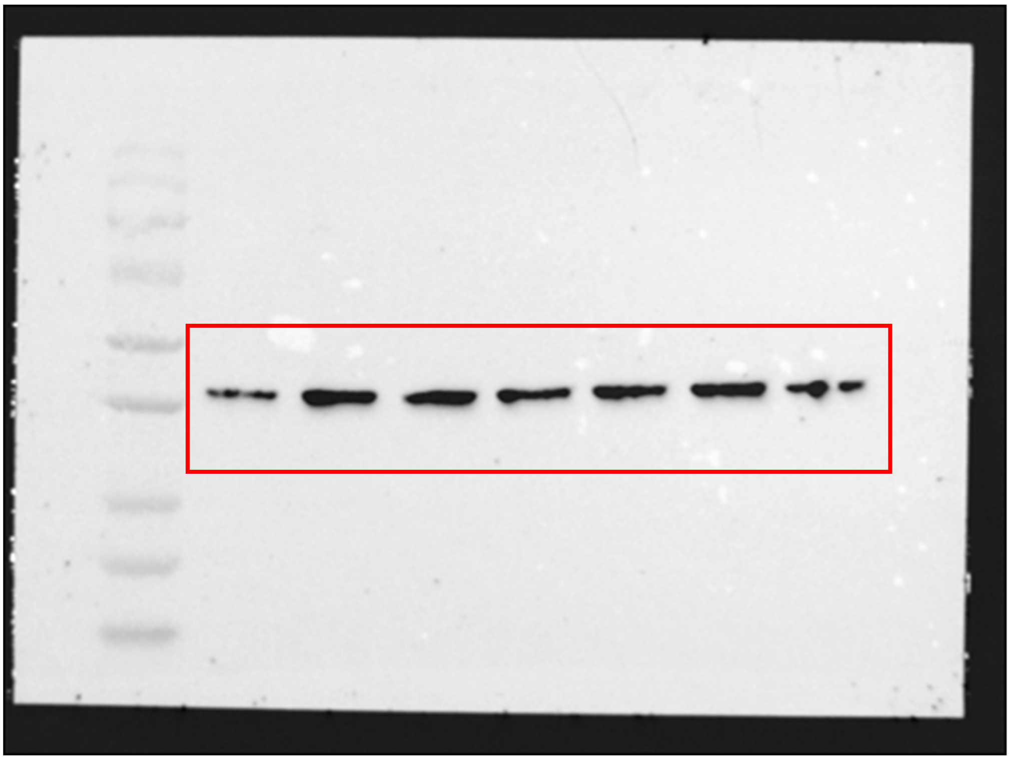

Uncropped Western blotting images. 36h Actin protein (Marked with red square).

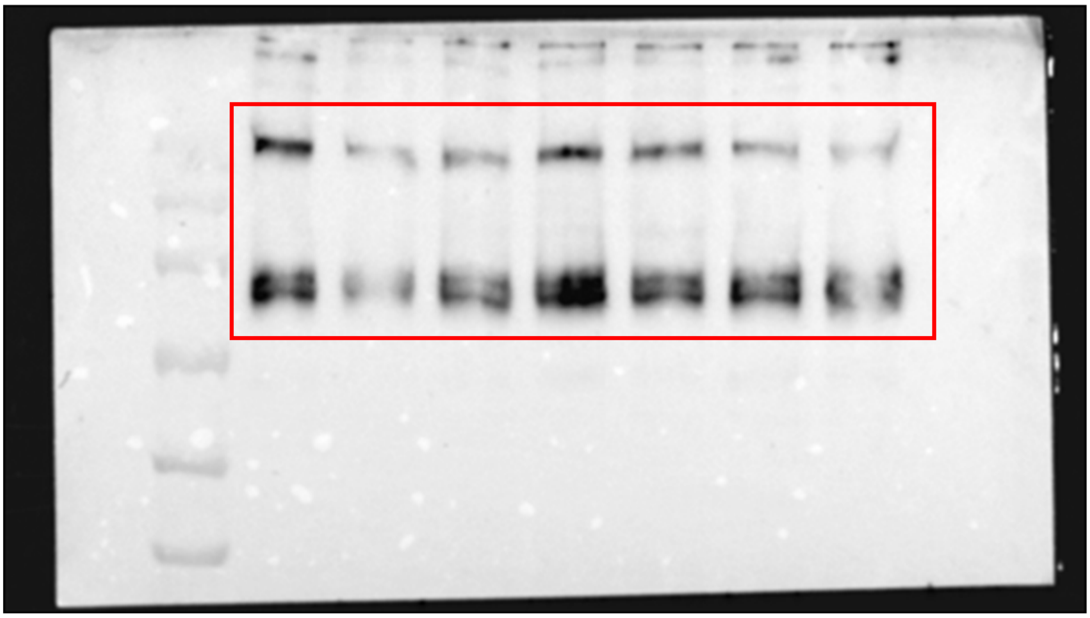

Uncropped Western blotting images. 36h S protein (Marked with red square).
